# “Exceptional brain aging” without Alzheimer’s disease: triggers, accelerators, and the net sum game

**DOI:** 10.1186/s13195-018-0373-z

**Published:** 2018-06-01

**Authors:** Prashanthi Vemuri

**Affiliations:** Department of Radiology, Mayo Clinic and Foundation, 200 First Street SW, Rochester, MN 55905 USA

**Keywords:** Exceptional Aging, AD prevention, Biomarker cascade

## Abstract

**Background:**

As human longevity increases and Alzheimer’s disease (AD) increasingly becomes a significant societal burden, finding pathways or protective factors that facilitate exceptional brain aging without AD pathophysiologies (ADP) will be critical. The goal of this viewpoint is two-fold: 1) to present evidence for “exceptional brain aging” without ADP; and 2) to bring together ideas and observations from the literature and present them as testable hypotheses for biomarker studies to discover protective factors for “exceptional brain aging” without ADP and AD dementia.

**Discovering pathways to exceptional aging:**

There are three testable hypotheses. First, discovering and quantifying links between risk factor(s) and early ADP changes in midlife using longitudinal biomarker studies will be fundamental to understanding why the majority of individuals deviate from normal aging to the AD pathway. Second, a risk factor may have quantifiably greater impact as a trigger and/or accelerator on a specific component of the biomarker cascade (amyloid, tau, neurodegeneration). Finally, and most importantly, while each risk factor may have a different mechanism of action on AD biomarkers, “exceptional aging” and protection against AD dementia will come from “net sum” protection against all components of the biomarker cascade. The knowledge of the mechanism of action of risk factor(s) from hypotheses 1 and 2 will aid in better characterization of their effect on outcomes, identification of subpopulations that would benefit, and the timing at which the risk factor(s) would have the maximal impact. Additionally, hypothesis 3 highlights the importance of multifactorial or multi-domain approaches to “exceptional aging” as well as prevention of AD dementia.

**Conclusion:**

While important strides have been made in identifying risk factors for AD dementia incidence, further efforts are needed to translate these into effective preventive strategies. Using biomarker studies for understanding the mechanism of action, effect size estimation, selection of appropriate end-points, and better subject recruitment based on subpopulation effects are fundamental for better design and success of prevention trials.

## Background

**Fig. 1:**
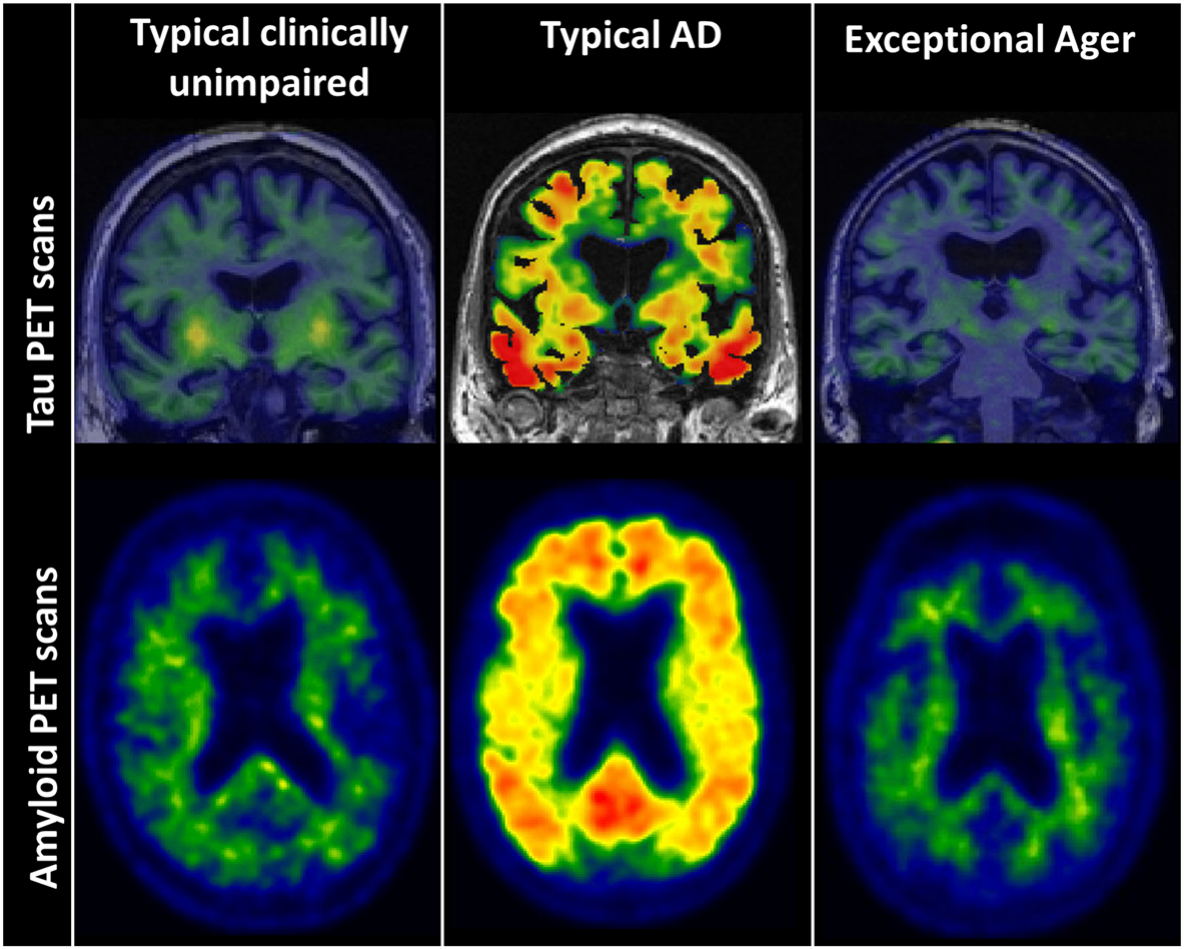
Tau and amyloid positron emission tomography (PET) scans in a typical clinically unimpaired, typical AD, and an exceptional ager (> 85-year-old APOE4 carrier)

The two primary histopathological changes to the brain due to Alzheimer’s disease (AD) are the deposition of amyloid and tau [1]. These two AD-related brain changes are the primary underlying causes of neurodegeneration and cognitive dysfunction which ultimately leads to dementia. As human longevity increases, and AD dementia increasingly becomes a major societal burden, finding pathways that lead to brain aging without AD pathologies (ADP) are critical. Currently, much of the research has been focused on resilience or cognitive reserve [2], wherein the focus has been on discovering how and why individuals are able to remain clinically unimpaired or cognitively normal despite ADP. However, it is important to investigate, using surrogates of amyloid and tau pathologies via cerebrospinal fluid (CSF) and positron emission tomography (PET), why majority of individuals develop ADP as they age and how some oldest old individuals are able to age without significant ADP. The latter individuals are called “exceptional agers” without ADP. While the absence of ADP can be defined using various thresholds, we refer to the absence of ADP as not reaching the neuropathological definition of AD in pathology studies and the imaging cutoffs of amyloid and tau positivity in imaging studies. Amyloid and tau PET scans of an exceptional ager in comparison to a clinically unimpaired individual and an AD dementia individual are shown in Fig. 1.

In this view point, the main goal is to bring together ideas and observations from the literature and present them as testable hypotheses or frameworks that can be employed in biomarker studies to discover protective factors or pathways to “exceptional brain aging”. In the context of the terminology we recently proposed, for hypotheses 1 and 2 the focus is on “resistance to ADP” [3] and for hypothesis 3 the focus is on both resistance to ADP and prevention of AD dementia.

These concepts are presented in the context of the primary AD pathophysiological processes in the biomarker cascade (amyloid, tau, and neurodegeneration due to AD pathologies). The focus is on primary prevention in midlife, designing effective trials by understanding the mechanisms of action on the biomarker cascade, and looking at the net sum protection against all components of the biomarker cascade. Although additional AD processes are not explicitly addressed, such as inflammation, synaptic and microglial dysfunction that are relevant to aging and AD dementia, the concepts here can also be extended to other measurable biomarkers that are mechanistically relevant to AD.

## “Exceptional brain aging” without ADP: is it really possible?

Several pathology and observational studies have provided evidence for aging without ADP [4, 5] and have focused on optimal or successful aging without cognitive decline [6–8] in the oldest old. In addition, specific evidence for “exceptional brain aging” without ADP comes from these three different lines of investigation.

### Prevalence of AD pathologies

**Fig. 2:**
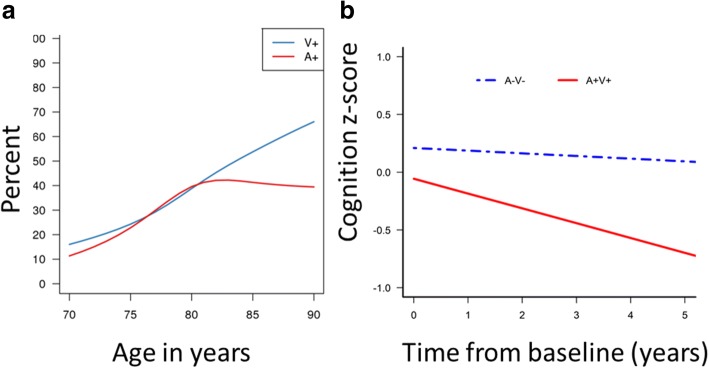
**a** Prevalence of elevated amyloid levels (A+) versus vascular disease (V+) in clinically unimpaired individuals based on data from Vemuri and Knopman [11]. Vascular pathologies show a steady increase in the prevalence (exponential growth curve models) but the amyloid positivity curves exhibit a slow saturation similar to logistic growth curve models. **b** Data from our previous study [25] illustrates the slow longitudinal cognitive decline seen in a clinically unimpaired 80-year-old male without amyloid and cerebrovascular pathologies (in blue) in comparison with significantly greater decline seen in a clinically unimpaired individual of the same age with both elevated amyloid and cerebrovascular pathologies (in red)

Nelson et al. [9] published an amalgamation of neuropathological literature showing that each added year of life does not lead to an increased prevalence of AD pathologies, unlike hippocampal sclerosis and cerebrovascular disease. Neuroimaging studies in the Mayo Clinic Study of Aging (MCSA) have also found non-monotonicity in the frequency of amyloid positivity in clinically unimpaired individuals [10, 11]. The data from our previous work [11] were consolidated to plot the prevalence of elevated amyloid versus excess cerebrovascular disease burden in clinically unimpaired individuals (Fig. 2a). These curves are reminiscent of two types of growth curve models in population ecology: exponential, or J-shaped, and logistic, or S-shaped, models. While exponential models have uninhibited growth in numbers, logistic growth models exhibit a slowing in growth as the population reaches its carrying capacity. Vascular pathologies show a steady increase in the prevalence or rate of growth representing an exponential model over an age range of 50–100 years. On the contrary, the amyloid elevation curves exhibit a slow saturation alluding to the fact that there may be a proportion of the population that will never develop elevated levels of amyloid, supported by evidence from Khachaturian et al. [12]. Amyloid data collected from 55 studies by Jansen et al. also showed that a logistic model was the best fit for amyloid prevalence [13].

### Declining AD incidence and amyloid levels

Recent evidence of age-specific decline in both incidence of dementia [14, 15] and amyloid levels [16] in aging brains provides compelling evidence for the possibility of aging without AD pathologies. With the strong possibility that better medical care and increasing education levels may have contributed to these declining trends [17], investigation into the underlying mechanisms may lead us closer to understanding the differences between normal aging and developing ADP.

### APOE4 carriers without AD dementia and AD pathologies in the oldest old

Age and the apolipoprotein (APO)E4 genotype are the two well-established risk factors for AD [13]. Therefore, one would expect that, as people age, the odds of an APOE4 individual developing AD dementia would increase with age. However, there have been several observations showing that the association between APOE4 genotype and development of AD dementia is weak in the oldest old, i.e., there are some APOE4 carriers who live into their 90s without AD dementia [12, 18–20]. While these studies have proven the presence of very old APOE4 carriers without AD dementia, one may argue that protection against AD dementia primarily comes from “resilience to ADP, i.e., coping with pathology”. However, the presence of amyloid-negative APOE4 cognitively normal individuals at 85 years of age (~ 25%) in a large meta-analysis [13] supports the idea of “resistance to ADP” in the oldest old APOE4 carriers.

While the observed evidence can be attributed to excess mortality early in life in those at risk (for example, for APOE4 carriers), it is important to study and understand how some individuals are able to age without ADP.

## Discovering pathways to “exceptional aging”

Given the possibility of “exceptional aging”, how does one discover the important protective factors. Three inter-related ideas or hypotheses are presented here that, when taken together, can aid in discovering protective pathways and help design effective preventive strategies.

### Hypothesis 1 (primary prevention in midlife)

*Discovering and quantifying links between risk factors and early ADP changes in midlife using longitudinal biomarker studies is fundamental to understanding why the majority of individuals deviate from normal aging to the AD pathway.*

#### Normal aging versus pathological aging

Aging acts through a number of biological mechanisms at the cellular or tissue level that lead to loss of reserve and function [21]. Prominent aging-related changes occur in the brain during midlife, and more so in the sixth to seventh decades. Midlife also represents the time during which (neurodegenerative and cerebrovascular) pathologies are observed in brain autopsies [9]. Even in the absence of pathologies, individuals suffer from age-related neural structure alterations [22, 23] and alterations in gene expression [24] starting in midlife. However, in the presence of neurodegenerative and cerebrovascular pathologies, the structural and functional deterioration of the brain has been observed to be greater. This accelerated decline in brain health due to neurodegenerative and cerebrovascular pathologies is the primary observed cause of dementia. By age 80, > 60% of clinically unimpaired individuals have either ADP or cerebrovascular disease. Figure 2b based on data from our previous study [25] illustrates the slow longitudinal cognitive decline seen in a clinically unimpaired 80-year-old male without amyloid and cerebrovascular pathologies (in blue) in comparison with a significantly greater decline in a clinically unimpaired individual of the same age with both amyloid and cerebrovascular pathologies (in red). There is also consensus about the significant heterogeneity in the cognitive aging process [7]. All these studies taken together provide evidence that normal aging is different from pathological aging and late midlife represents a critical time period during which we observe noticeable divergence of these two pathways. Given that slowing of age-related changes in midlife can be observed with better lifestyle factors such as physical activity and ideal levels of cardiovascular health [26–28], our focus should be on primary prevention during midlife and early adulthood.

There is well-established literature supporting that midlife conditions have a significant impact on late-life dementia, especially cardiorespiratory fitness [29] and vascular risk factors [30]. The relationship between several risk factors (obesity, hypertension, dyslipidemia) and dementia incidence has been observed to be U-shaped in nature with the greatest association during midlife [31–33]. Additionally, the prevalence of amyloid curves (as mentioned above) follows a logistic growth curve model with the greatest rate of amyloid accumulation in the population during late midlife. The first hypothesis proposes that greater focus needs to be placed on longitudinal biomarker studies that can discover and quantify links between risk factors in midlife and increased ADP accumulation in late midlife to understand why individuals deviate from the normal aging process.

One may argue that there has been extensive literature already supporting the hypothesis that midlife risk factors such as vascular risk factors increase late life dementia incidence. However, the results from intervention studies based on a reduction of vascular risk factors [34] highlights the need for longitudinal biomarker studies in midlife that focus on understanding the mechanisms of action of the suggested risk factors as early ADP changes evolve. This is especially important for risk or protective factors that are highly debated in the literature [35–37]. Understanding how the risk factors or combination of risk factors impact early ADP changes (whether it is amyloid, tau, or neurodegeneration) using longitudinal studies will facilitate a better understanding of how protective factors can be employed for primary prevention [38, 39]. While significant focus has been placed on amyloid imaging since it has been available from the mid-2000s, the same concepts can be extended to tau-related studies as longitudinal tau data become available [40].

### Hypothesis 2 (designing effective trials)

*A specific risk factor may have quantifiably greater impact as a trigger and/or accelerator on a specific component of the biomarker cascade (amyloid, tau, or neurodegeneration).*

#### The biomarker cascade framework and quantifying the impact of each risk/protective factor

Although amyloid and tau deposition can be initiated independently, there is sufficient recent evidence supporting the hypothesis that amyloid deposition accelerates tau deposition which, in turn, is closely associated with cognitive decline [41–44]. Autosomal dominant AD studies that represent younger-onset pure AD cases have confirmed the sequence of amyloid followed by tau, followed by cognitive decline [45, 46]. The biomarker model presented and refined based on the literature by Jack et al. [43] synthesized AD processes into a set of testable hypotheses. Amyloid, tau, neurodegeneration, and cognitive decline form the biomarker cascade and this framework has helped significantly improve our understanding of disease onset and progression [41, 47–49].

The presence of suspected non-AD pathophysiology (SNAP; neurodegeneration in the absence of amyloid) [50] and primary age-related tauopathy (PART) in the absence of amyloid [51] illustrate the heterogeneity in the age-related neurodegenerative processes and share some pathophysiological aspects (neurodegeneration or tau) of the AD biomarker cascade. Since each of these pathophysiologies plays a role in the development of AD dementia, as discussed further in hypothesis 3 below, studying independent triggers and accelerators for each component of the AD biomarker cascade will be important. In the second hypothesis, it is proposed that looking at each individual component of the biomarker cascade (amyloid, tau, neurodegeneration) to explore the impact of the risk factor of interest will aid in understanding the mechanisms through which the specific risk factor impacts AD processes.

#### Importance of knowing the mechanisms

Although a vast amount of literature has provided evidence for the impact of risk factors on dementia incidence, less has been published on the impact of each individual risk factor on the primary disease mechanisms. Discerning the disease stage at which the reduction of a specific risk factor would be helpful will be important for designing effective preventive strategies. A recent example was the failure of the TOMORROW trial, which targeted diabetes medications for reduction of dementia [38]. While there has been substantial evidence that diabetes is associated with AD dementia incidence, the primary mechanism of action may be through neurodegeneration (discussed further below) [52]. Therefore, with diabetes as a preventive strategy, the focus should be on measuring the reduction in neurodegeneration and not on reduction in amyloid deposition. Another example is that of sleep as a preventive strategy. While poor sleep has been shown to impact amyloid deposition through poor clearance of amyloid [53, 54], and thus could mechanistically be linked to greater dementia incidence [55] and brain atrophy [56], improving sleep quality as a preventive strategy for AD dementia may fail in individuals who have high levels of amyloid. Therefore, quantifying the effect size of risk factors on each component of the biomarker cascade will aid in choosing appropriate outcomes and the sample sizes required. In addition, determining the effect modifiers (main biological and disease-related factors that may influence the treatment response such as additional interactions of the risk factors with age and APOE4 status) will aid in better enrichment strategies and intervention optimization.

**Fig. 3:**
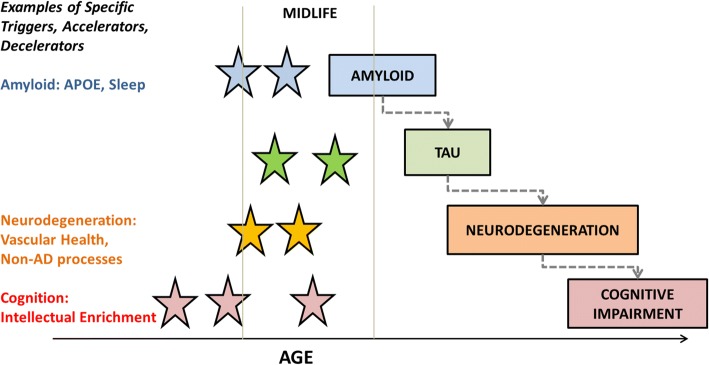
Framework for the second hypothesis and examples of triggers and accelerators of some of the components of the biomarker cascade. AD, Alzheimer’s disease; APOE, apolipoprotein E

Figure 3 illustrates well-established triggers and accelerators for some of the components of the biomarker cascade. A specific example of vascular health and neurodegeneration is discussed here. Poor vascular health and vascular risk factors are clearly related to higher incidence of dementia [57] as well as causing significant brain changes independent of amyloid and tau [58]. While there has been no doubt that vascular risk factors, specifically diabetes and hypertension, increase neurodegeneration (cortical thinning and hippocampal atrophy), there has been considerable debate about the impact of vascular risk on amyloid deposition. In a recent study, we found that the impact of vascular health was quantifiably greater on neurodegeneration than on amyloid deposition supporting the second hypothesis [52]. If one were to consider that vascular risk factors cause significantly greater neurodegeneration and cognitive decline compared with their effect on early amyloid deposition, it strongly supports the epidemiological findings that vascular risk factors lower the threshold of dementia detection and are related to a higher incidence of dementia [57].

### Hypothesis 3 (net sum game)

*“Exceptional aging” as well as protection against AD dementia will come from “net sum” protection against all the components of the AD biomarker cascade.*

If protection against AD pathology in each individual were viewed as a “net sum” of effects from all triggers and accelerators (lifestyle, midlife risk factors, chronic conditions, net difference between protective and risk genes) as well as additive and interactive non-AD processes, then “exceptional aging” without ADP and ultimately without AD dementia would be possible if a large positive “net sum” were present. This hypothesis highlights the importance for multifactorial or multidomain approaches to “exceptional aging” without ADP and AD dementia.

**Fig. 4:**
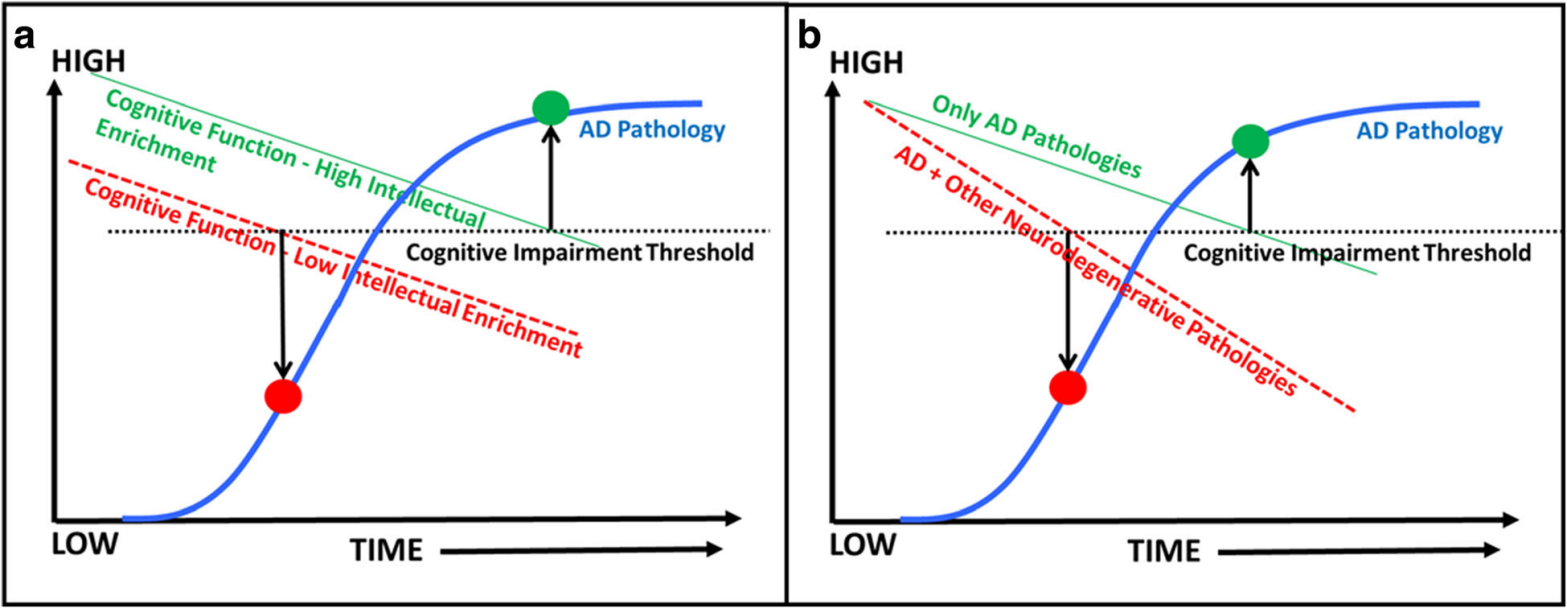
Impact of intellectual enrichment (e.g., education, occupation, cognitive activity) and Other neurodegenerative processes on the Alzheimer’s disease (AD) trajectories. Cognition curves are superimposed on the ADP curves (amyloid or tau) shown in blue. The horizontal line indicates the cognitive impairment threshold. The time at which cognitive function meets the threshold allows us to deduce the ADP levels at the same time point on the superimposed ADP curves. **a** Illustration of individuals with high (green curve) and low (red dashed line) intellectual enrichment and the ADP levels (shown by the circles) at which cognitive impairment would be observed in both groups of individuals. **b** Illustration of individuals with only AD path (green curve) and AD path in addition to other neurodegenerative pathologies (red dashed line) and the ADP levels (shown by the circles) at which cognitive impairment would be observed in both groups of individuals

The support for “net sum” against AD dementia primarily comes from dementia risk score studies [59, 60] that have shown that a combination of several risk factors are best at predicting dementia risk compared with individual risk factors. The large positive “net sum” against ADP was also observed in our recent study where we found that, irrespective of the impact of a risk factor on amyloid or neurodegeneration, several protective factors (absence of midlife risk factors, lower chronic conditions) had moderate effect sizes in predicting those who were greater than or equal to 85 years of age without abnormal amyloid and neurodegeneration levels compared with those who had significant amyloid and neurodegeneration [37]. In addition, greater intellectual enrichment can further aid in delaying the onset of impairment through its impact on cognition, as illustrated by Fig. 4a [61–63].

The presence of non-AD processes such as cerebrovascular disease, TDP-43, Lewy bodies (often alongside AD processes) and their contribution to cognitive impairment are important to consider in this context since non-AD neurodegenerative pathologies reduce the threshold to AD dementia when present along with ADP [57, 64]. This concept can be observed in Fig. 4b, which illustrates two subsets of individuals: the first have cognitive decline or neurodegeneration only due to ADP, and the second have a greater rate of neurodegeneration or cognitive decline due to other non-AD neurodegenerative processes along with ADP. A clear difference can be observed in the levels of ADP at which the same level of cognitive impairment would be expected for both groups. The second group would need a much lower level of amyloid to experience the same level of cognitive impairment as the first group. This figure illustrates the importance of viewing protection against AD dementia as protection against all components of the AD biomarker cascade.

A major limitation of this work was limiting the scope to the three main AD biomarkers for simplicity. However the concepts illustrated in Figs. 3 and 4 can be extended after inclusion of additional measurable AD-specific processes such as inflammation as well as non-AD processes and pathologies.

## Conclusions

While important strides have been made in identifying risk factors for AD dementia incidence, future efforts need to be directed towards discovering the timing and mechanism of action of each of these risk factors on AD processes. In this work, three inter-related ideas are presented that are important to consider while studying risk factors and may help us move towards developing effective preventive strategies to maneuver individuals away from the AD pathway towards the pathway of “exceptional brain aging” without ADP.
